# A U-Net model for epidermal segmentation in optical coherence tomography images of actinic keratosis

**DOI:** 10.1371/journal.pone.0346059

**Published:** 2026-06-05

**Authors:** Theofanis Angelis, Peter A. Philipsen, Vinzent K. Ortner, Gabriella Fredman, Merete Haedersdal, Gavrielle R. Untracht

**Affiliations:** 1 Department of Dermatology, Copenhagen University Hospital, Bispebjerg and Frederiksberg, Copenhagen, Denmark; 2 Department of Health Technology, Technical University of Denmark, Kongens Lyngby, Denmark; 3 Department of Clinical Medicine, Faculty of Health and Medical Science, University of Copenhagen, Copenhagen, Denmark; Bayer Crop Science United States: Bayer CropScience LP, UNITED STATES OF AMERICA

## Abstract

Actinic keratosis (AK) is a pre-cancerous skin lesion typically caused by excessive exposure to ultraviolet light. Optical coherence tomography (OCT) can provide sub-surface information relevant for lesion classification, but manual analysis of images is time-consuming and subject to inter-observer variability; automated segmentation based on deep learning models can provide faster and more consistent results. However, structural changes, such as thickening and hyperkeratosis, complicate epidermal segmentation tasks. For this reason, we aimed to develop and optimize a U-Net model for the automated epidermal segmentation of AK lesions in OCT images, combining both accuracy and computational efficiency. Multiple configurations were evaluated by varying hyperparameters, including image sizes (256 × 256, 512 × 512, 1024 × 1024, and 464 × 1356 pixels), batch sizes (2, 4, 8, and 16), and training durations (50, 100, and 150 epochs). Model performance was evaluated quantitatively using several metrics including the Dice coefficient and Jaccard index, comparing automated epidermal segmentations against expert annotations. The optimal configuration, with an image resolution of 256 × 256 pixels and a batch size of 2 over 50 epochs, had a Dice score of 0.86 and a Jaccard index of 0.76. Higher resolutions and longer training increased computation without significantly enhancing the accuracy values and sometimes caused overfitting. These findings show that a simple U-Net architecture could achieve efficient and accurate epidermal segmentation in AK lesions, provided it is fine-tuned to enhance performance.

## 1. Introduction

Actinic keratosis (AK) is a common pre-cancerous skin lesion that may develop into squamous cell carcinoma (SCC) if it remains untreated [1]. It appears as solitary or multiple rough, scaly patches on sun-exposed areas like the face, scalp, and hands. Not all AKs become malignant; however, many SCCs arise from these lesions [2]. Therefore, early detection and precise assessment are crucial for preventing invasive skin cancer.

Traditional clinical evaluation of AK lesions, mainly relying on visual inspection with or without dermatoscopy, is subjective and highly dependent on the clinician’s expertise. In the case that SCC is suspected, histopathology may be used to assess the integrity of the dermal-epidermal junction (DEJ), as invasion into the dermis is a key distinguishing feature of SCC [3,4]. While histopathology provides definitive diagnosis, it requires invasive biopsies [5,6]. Additionally, it provides tissues structure only at the location of the biopsy and does not provide information about surrounding tissue. Other non-invasive imaging methods, such as reflectance confocal microscopy and high-frequency ultrasound, have been explored to improve diagnostic accuracy, but they are limited in terms of imaging depth or resolution [7,8]. Diagnosis of AK should primarily rely on clinical examination supported by non-invasive imaging techniques, with biopsy reserved for clinically or dermatoscopically suspicious or treatment-refractory lesions, according to the 2024 European consensus-based interdisciplinary guideline [9].

Optical coherence tomography (OCT) is a non-invasive imaging modality that utilizes light to provide high-resolution, real-time, cross-sectional images of tissue microstructure up to 1–2 mm deep in skin [10]. It is a valuable tool offering objective structural information without the need for biopsy, allowing the visualization of the epidermis, dermis, DEJ integrity, and lesion morphology. However, manually analyzing and annotating OCT images is time consuming, and annotations can vary depending on the individual performing the work. Also, although OCT is mentioned in the new European guidelines for diagnosis of AK [9], interpretation of OCT images is not included in the clinical training curriculum; this is a significant barrier to the widespread adoption of OCT in dermatology. Hence, there is a real need for automated tools that can help clinicians assess AK more efficiently, promptly, and reliably [11]. In particular, automated segmentation of the DEJ in AK could help with lesion grading, risk profiling, and confirming the diagnosis of AK vs SCC noninvasively.

The automated segmentation of AK lesions is not a straightforward process. AK lesions are heterogeneous, varying between patients and across body areas, and can appear with no consistent pattern [12]. Several different classification schemes exist for evaluating AK lesions. Clinically, AK lesions are commonly classified according to the Olsen grading scheme, which grades lesions based on the lesion thickness and presence of hyperkeratosis: grade 1 corresponds to mild, grade 2 to moderate, and grade 3 to severe lesions with pronounced hyperkeratosis [13]. While no correlation has been found between the Olsen grade and the risk of progression to SCC [14,15], structural changes caused by AK, including epidermal thickening and differences in lesion grades (1-3), complicate automated segmentation, with higher-grade lesions being generally more challenging to segment.

Although the automated segmentation of AK can be challenging due to morphological factors and limited imaging depth, deep learning models have shown effectiveness in overcoming these difficulties [16,17]. U-Net is a convolutional neural network (CNN) model that can capture both the entire image context and fine structural details due to its encoder–decoder design with skip connections [18]. Compared to other deep learning models such as fully convolutional networks (e.g., V-Net), or more recent transformer-based approaches, U-Net is widely used because of its good performance with relatively small datasets. It provides accurate boundary localization with computational efficiency for pixel-level prediction tasks [19]. However, its results depend strongly on the appropriate choice of parameters, such as image size, batch size, and training time [20].

In our study, we developed a U-Net model specifically optimized for segmentation of the epidermis in OCT images of AK. We sought to strike a balance between accuracy and efficiency, aiming to minimize computation time. We performed a quantitative analysis across different hyperparameter configurations to compare automated annotations with expert manual annotations. Overall, our findings shed light on how deep learning can be effectively implemented in OCT workflows to support faster, more robust assessment and monitoring of AK.

## 2. Methods

### 2.1. Dataset

The dataset comprised retrospective data from the Copenhagen Actinic Keratosis Study (COAKS), a single center, randomized clinical trial undertaken at the Department of Dermatology, Copenhagen University Hospital, Bispebjerg, Denmark [21]. Adults with 4–8 AK lesions with Olsen grade 1–2 in a 5 × 5 cm area on the face, neck, or chest were included in the study. In total, 60 participants were enrolled in the study. The trial was authorized by the Danish Medicines Agency (2021032485) and the Capital Region of Denmark’s Ethics Committee (H-21018064) and registered on clinicaltrials.gov (NCT05164393). The trial was monitored in accordance with the International Council for Harmonization (ICH) Good Clinical Practice guideline and all applicable regulatory requirements. Written informed consent was obtained from all participants in adherence to the Declaration of Helsinki. The dataset was accessed between March – June 2025. The authors did not have access to any information that could identify individual participants at any time.

OCT images were acquired using the VivoSight Dx system (Michelson Diagnostics Ltd, Maidstone, Kent, UK). This system is a swept-source OCT system, offering a field of view of 6 mm × 6 mm, with axial and transverse resolutions of 5.5 µm and 7.5 µm, respectively, in air at a 20 kHz line scan speed. Volumetric images were collected over a 6 mm × 6 mm area, using sampling intervals of 4.4 μm along the fast axis and 41 μm along the slow axis. Each AK lesion was imaged using an OCT volume scan, consisting of 120 B-scans.

In total, 984 OCT volumes were acquired across three different time points during the study, of which 317 were baseline scans of AK lesions. Only baseline scans of AKs, captured before any treatment, were included in this study. These baseline scans were obtained from all 60 patients and included lesions on the face, neck, and chest. To train and evaluate the deep learning model, a representative subset of 60 baseline scans was randomly selected, and the central (middle) B-scan from each volume was chosen as the representative image. The central B-scans were chosen for primary analysis, as they are generally considered representative of the lesion’s overall structure and thickness. These slices capture the maximal cross-section along the slow axis and contain the most clinically relevant features for segmentation. To further evaluate the impact of slice location and strengthen our primary choice, we conducted an additional experiment where our trained model was applied to both non-central (at 25% and 75% peripheral positions) and central (50%) B-scans on an additional dataset from multiple patients and body sites (chest, temple, forehead), assessing robustness along the slow axis. Automated U-Net segmentation masks were compared against manual masks across 15 B-scans covering these positions using the evaluation metrics described below. Significant differences in the metrics were assessed using a paired t-test with significance set to p < 0.05.

### 2.2. U-Net architecture

The proposed model is a U-shaped, fully CNN for epidermal image segmentation. It is based on the well-known U-Net architecture proposed by Ronneberger et al. [18], with several modifications tailored to the specific segmentation task and designed to reduce computational complexity. The network follows a symmetrical encoder-decoder structure, where corresponding layers in the encoder and decoder are connected through skip connections. The encoder compresses the image, focusing on the big picture, while the decoder rebuilds the details, using high-resolution information from the encoder. This setup helps the model produce accurate segmentation maps. Additionally, it performs well even with a small number of labeled images [20].

**Fig 1:**
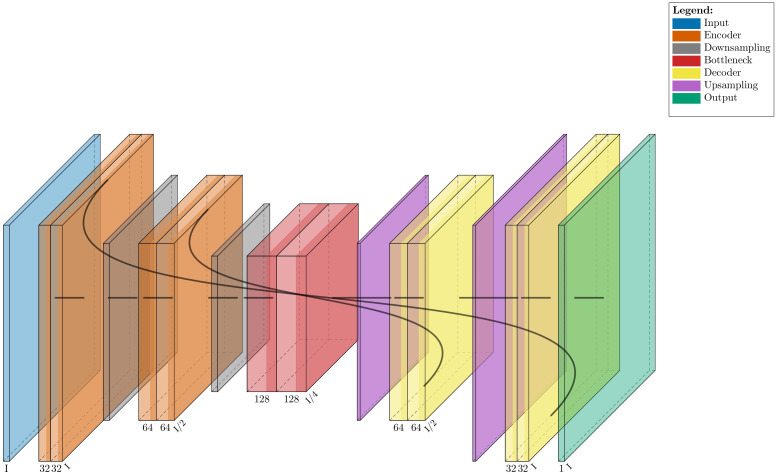
The proposed U-Net schematic with the encoder-decoder layout and skip connections [25]. The network consists of an input layer (blue), an encoder with two convolutional blocks (orange), a bottleneck layer (red), a decoder with two convolutional blocks (yellow), and an output layer (green). Gray blocks and purple represent downsampling and upsampling operations, respectively. Skip connections are shown linking encoder and decoder layers. “I” represents the spatial size of the input image.

To enhance the model’s efficiency and performance, we made several modifications to the conventional U-Net architecture. Instead of using the typical (3 × 3) filters for upsampling, smaller (2 × 2) filters were chosen to make the process more efficient without losing important features [22]. To ensure the output remains the same size as the input, we used “same” padding, which adds zero padding around the image [23]. This helps keep the details, especially at the edges, intact. We also built the model using Keras instead of Caffe because Keras is more user-friendly and intuitive [24]. Fig 1 provides an overview of the model’s structure [25].

### 2.3. Training and testing strategy

The U-Net model was implemented using Python 3.8.20 with the TensorFlow and Keras libraries 2.12.0, within the Anaconda environment. All training and testing of the U-Net model were performed using only a CPU. The 60-image dataset was randomly divided into 80% for training (48 images), which included both training and internal validation, and 20% for testing (12 images). The same image sets were used for training and testing all models. Internal validation and the Keras’ built-in validation were the key mechanisms to monitor loss, apply EarlyStopping, and adjust the learning rate in the training phase. No test images were used during training or validation. The test set was evaluated only once after training to identify the most optimal U-Net model.

**Fig 2:**
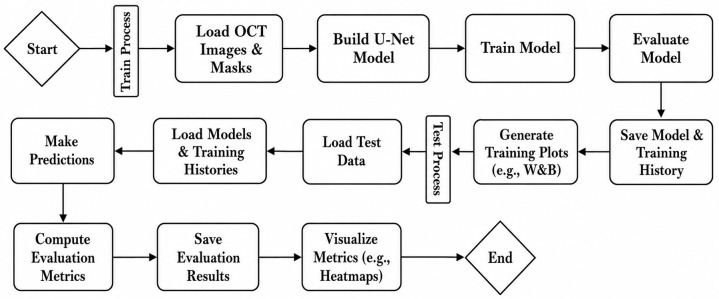
Model training/testing pipeline. Workflow of the U-Net model, including data loading, model training, testing, evaluation, and result visualization.

Manual segmentation of the epidermis was performed by a medical doctor with experience in OCT imaging (GF), using ImageJ software (US National Institutes of Health, Bethesda, Maryland, USA). These manual annotations served as ground truth for generating the segmentation masks used in model training and evaluation. The complete process is illustrated in Fig 2, which shows the model training and testing pipeline, including data loading, model training, testing, evaluation, and result visualization.

**Fig 3:**
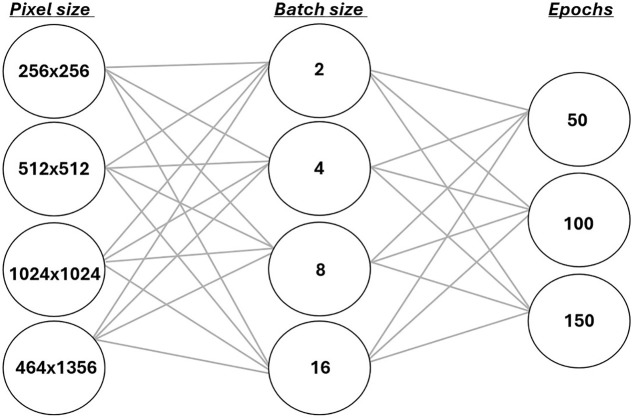
Configuration parameters, including image sizes in pixels (pixel sizes—in the left column), batch sizes (middle column), and epochs (right column). A total of 36 different hyperparameter configurations were tested.

The proposed U-Net model was trained using the Adam optimizer at a learning rate of 0.001 [20]. All experiments were conducted on an HP EliteDesk 805 G6 Small Form Factor PC, equipped with an AMD Ryzen 5 PRO 4650G processor featuring Radeon Graphics (3.70 GHz), 64 GB of RAM (63.3 GB usable), and running Windows 11. Fig 3 illustrates the various input hyperparameter combinations, specifically sets of image sizes in pixels, batch sizes, and training epochs, to thoroughly investigate image segmentation through these configurations.

### 2.4. Evaluation metrics

The optimization and the effectiveness of the trained U-Net models were evaluated using several metrics, including accuracy, precision, recall, Jaccard index (also called intersection over union (IoU)), Dice coefficient, mean absolute error (MAE), and Hausdorff distance (dH), to compare the predicted segmentation masks with the manually annotated ground truth [26,27]. Table 1 presents overview of the metrics used in this study, their definitions, and interpretations. Accuracy, precision, recall, IoU, and Dice coefficient all provide a unitless score in the range [0–1], where higher numbers (closer to 1) indicate better segmentation performance;  > 0.7 is considered acceptable, > .8 is good, and >0.93 is excellent [28]. MAE and Hausdorff distance are in units of pixels and fall within the range of [0,∞) where 0 indicates perfect segmentation. Although they depend on factors such as the image size in pixels, we would define acceptable ranges based on our data. Hence, MAE < 1 pixel and dH < 25 pixels are considered a good performance range, whereas a MAE between 1–1.5 pixels and a dH between 25–70 pixels are considered acceptable for model performance across our proposed different image resolutions.

**Table 1:** Evaluation metrics for segmentation tasks.

Metric	Formula	Explanation & Relevance
Accuracy	$\frac{TP+TN}{TP+TN+FP+FN}$	Overall rate of correct pixels. Less useful when the number of background pixels is much larger than the number of foreground pixels.
Precision	$\frac{TP}{TP+FP}$	Amount of correctly predicted epidermis pixels. Checks for over-segmentation.
Recall	$\frac{TP}{TP+FN}$	Amount of true epidermis pixels were found. Checks for missed regions.
Jaccard index (IoU)	$\frac{TP}{TP+FP+FN}$	Overlap between prediction and ground truth. Balanced overall score.
Dice coefficient	$\frac{\text{2}\cdot TP}{\text{2}\cdot TP+FP+FN}$	Similar to IoU, but more sensitive to thin structures like the epidermis.
Mean absolute error (MAE)	$\frac{\text{1}}{N}\sum_{i=\text{1}}^{N}\left|y_{i}-{\hat{y}}_{i}\right|$	Average of the absolute difference between prediction and ground truth. Indicates overall prediction error.
Hausdorff distance (dH)	$max\left(\begin{array}{c} max\\ p_{A}\epsilon L_{A} \end{array}\begin{array}{c} min\\ p_{B}\epsilon L_{B} \end{array}d\left(p_{A},p_{B}\right),\begin{array}{c} max\\ p_{B}\epsilon L_{B} \end{array}\begin{array}{c} min\\ p_{A}\epsilon L_{A} \end{array}d\left(p_{A},p_{B}\right)\right)$ where L_A_ & L_B_: predicted & ground truth mask boundaries, p_A_ ∈ L_A_ & p_B_ ∈ L_B_ are points on them, d(p_A_,p_B_): Euclidean distance between these points, with min d(p_A_,p_B_) representing the shortest distance from a predicted point to the ground truth boundary.	Largest boundary mismatch. Measures the worst-case boundary deviation between prediction and ground truth. Important for precise epidermal thickness and shape.

TP: True Positives, FP: False Positives, FN: False Negatives, TN: True Negatives, y_i_: manual annotated masks (ground truth), ŷ_i_: U-Net predicted masks, and N is the total number of pixels being compared.

To evaluate the clinical relevance of the segmentation performance, epidermal thickness (ET) was computed from both manual and automated epidermal segmentations. For each central B-scan of our test dataset, ET was calculated as the mean vertical distance between the upper and lower epidermal boundaries across image columns. Agreement between manual and automated measurements was evaluated using correlation (scatter plot) and Bland–Altman analyses.

## 3. Results

### 3.1. Training and validation performance of U-Net models

**Fig 4:**
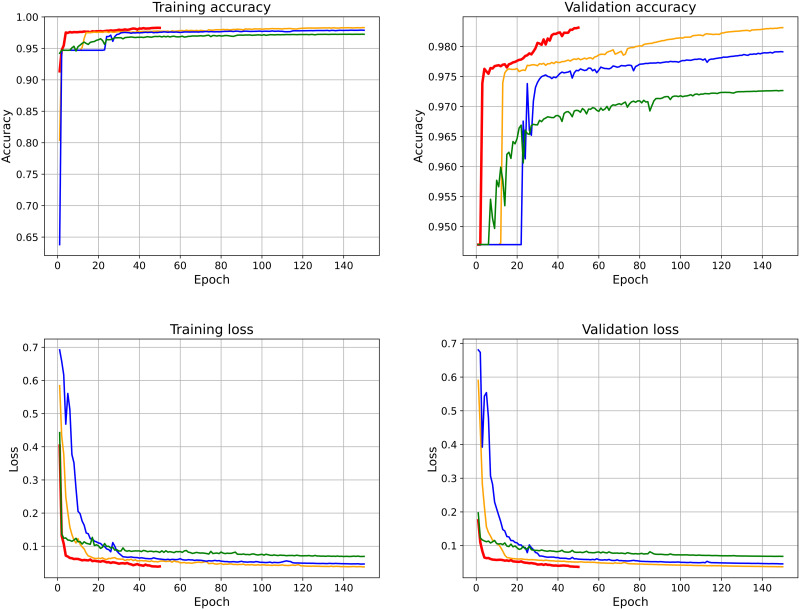
Training and validation accuracy and loss curves for selected U-Net models. The optimal setup used pixel size of 256 × 256 and a batch size of 2 with 50 epochs (or *PS*256 × 256-B2-E50), marked by a thick red line. Other configurations present overfitting resulting from prolonged training (*PS*256 × 256-B8-E150, shown in orange), suboptimal performance with large batch size (*PS*256 × 256-B16-E150, shown in blue), and increased computational demands associated with higher resolution (*PS*1024 × 1024-B2-E150, shown in green).

The training and validation accuracy and loss for selected U-Net models, each tested with different hyperparameters, are shown in Fig 4. In all, 36 combinations of hyperparameters were selected; the full list can be found in S1 Table. Training loss and accuracy show how well the model fits the training data, while validation loss and accuracy indicate the performance of the model on new data that was not used during training, i.e., the validation dataset. The curves for all tested models can be found in S1 Fig. Most of the models achieved a high accuracy greater than 95%. However, some models showed signs of overfitting; although the training accuracy remained high, the validation accuracy decreased, and the validation loss was higher than the training loss. Also, a fully detailed table listing all tested configurations (image size, batch size, epochs, and whether they converged or overfit) is included in S2 Table. The optimal model, highlighted in red, used a 256 × 256 pixels input image size and a batch size of 2 over 50 epochs. It showed strong results on both the training and validation sets, with the lowest validation loss. Increasing the input size to 512 × 512 pixels did not significantly improve performance, but it did make the training process slower and more resource-intensive. On the other hand, the use of smaller batch sizes allowed the model to be updated more frequently, thereby avoiding poor solutions and improving validation performance.

**Fig 5:**
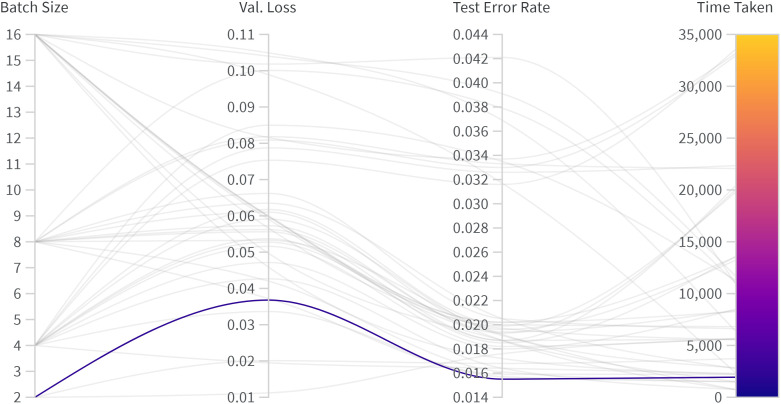
Weights & biases analysis showing the relation between batch size, validation loss, test error rate, and time spent for the U-Net configurations. The dark purple line illustrates the configuration leading to the most optimal performance.

To better understand the influence of hyperparameters, the optimization process was monitored using a weights & biases analysis to compare model performance. Fig 5 shows a parallel coordinates chart that summarizes the relationships between batch size, validation loss, test error rate, and training time. The analysis confirms the earlier findings, specifically that smaller batch sizes (especially a batch size of 2) tend to produce lower validation loss and test error rates, albeit with slower training convergence.

### 3.2. Comparison of manual and automated segmentations

Each of the performance metrics was calculated for each hyperparameter configuration; a full overview of the metrics is shown in S1 Table. The setup of image size of 256 × 256 pixels with a batch of 2 over 50 training epochs showed the best overall performance, achieving the highest Dice coefficient (0.859), Jaccard index (0.762), recall (0.839), and accuracy (0.985), closely matching expert annotations. At a resolution of 256 × 256 pixels, the model trained with a batch size of 4 over 100 epochs had the lowest Hausdorff distance (10.432) and the model trained with a batch size of 8 over 50 epochs achieved the highest precision (0.924) and lowest MAE (0.134) Table 2. These results emphasize that our U-Net model is functional and robust for small datasets at image size of 256 × 256 pixels, particularly with small batch sizes and moderate training durations. Higher image sizes and longer training time did not result in a clear performance improvement and sometimes led to overfitting or reduced generalization.

**Table 2:** Performance of selected U-Net models on the testing set.

U-Net Model Name	Accuracy	Precision	Recall	Jaccard Index	Dice Coefficient	Mean Absolute Error	Hausdorff Distance
**PS_256 × 256_B2_E50**	**0.985**	0.891	**0.839**	**0.762**	**0.859**	0.214	12.026
PS_256 × 256_B4_E100	0.984	0.887	0.836	0.757	0.856	0.241	**10.432**
PS_256 × 256_B8_E50	0.981	**0.924**	0.729	0.687	0.808	**0.134**	13.612
PS_256 × 256_B8_E150	0.984	0.890	0.829	0.753	0.853	0.234	11.922

The best-performing values are bold and the most optimal configuration (PS_256 × 256_B2_E50, corresponding to image size 256 × 256 pixels, batch of 2 over epochs 50) is marked through the corresponding metrics. PS: image size in pixels, B: batch size, E: epochs.

**Fig 6:**
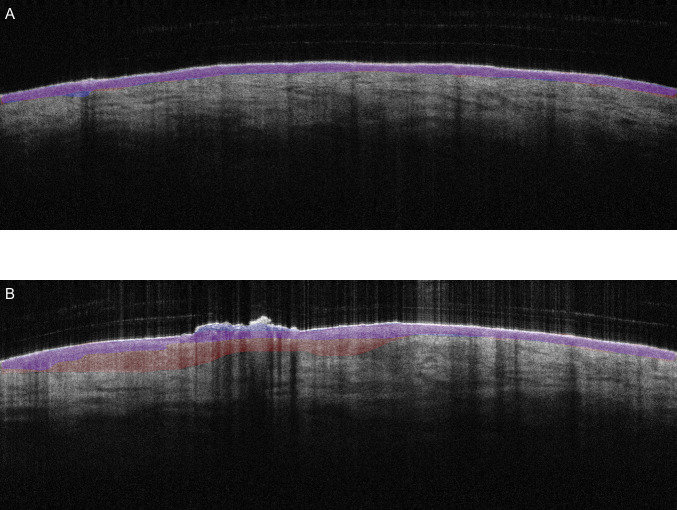
Segmentation examples showing high (A) and low (B) agreement between manual (red) and automated (blue) epidermal masks. Blue masks were generated by the U-Net model (image size 256 × 256 pixels, batch size 2, 50 epochs). In both, purple color indicates the overlay between them.

Fig 6 displays the segmentation results on two test cases within the testing set, comparing manual and automated masks generated by the optimal U-Net configuration. Fig 6A illustrates a case with close agreement between manual and U-Net automated segmentations, where the U-Net predicted mask accurately follows the epidermal boundary. In contrast, Fig 6B presents an example where the automated segmentation differs from the manual annotation.

In most cases, the manual annotations and the proposed U-Net automation are closely aligned, especially in regions with high signal quality and clearly defined epidermal boundaries. Some discrepancies were observed in areas where the boundaries of the dermal-epidermal (DEJ) may be poorly defined or have poor contrast. To assess the generalizability of our proposed epidermal segmentation model, we evaluated its performance on both central and peripheral B-scans within the imaging volume. Additional details and clarifications on the evaluation of B-scan regions and statistical comparison are provided in S1 File. The evaluation metrics, including Dice, IoU, precision, recall and accuracy, are shown in S3 Table for peripheral (25% and 75%) and central (50%) regions. Dice and IoU scores on these peripheral slices were comparable to those on central B-scans, with no statistically significant differences (all p > 0.05). These findings show that the model also performs well to non-central slices, highlighting robustness along the full lesion volume (see S4 Table for full pairwise comparisons).

**Fig 7:**
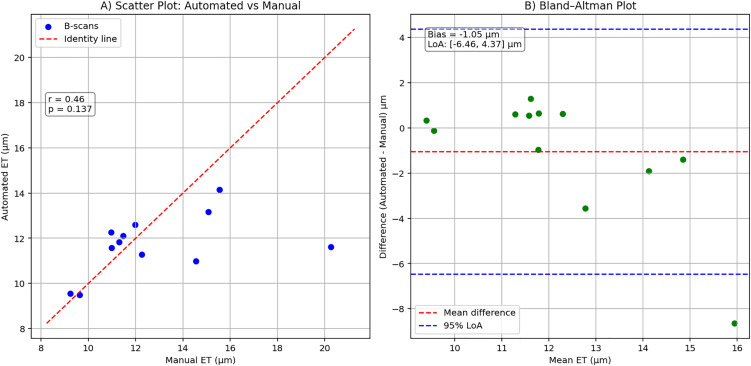
Comparison of automated and manual epidermal thickness. **(A)** Scatter plot of automated vs manual epidermal thickness (ET) measurements for each of the testing data. The red dashed line shows perfect agreement; correlation was moderate (r = 0.46, p ≈ 0.14). **(B)** Bland–Altman plot of the difference between automated and manual ET measurements vs their mean. The red dashed line shows the mean difference (Bias, –1.05 µm) and blue dashed lines indicate 95% limits of agreement (LoA, –6.46 to 4.37 µm).

Additionally, to assess the clinical applicability of our model, automated ET measurements were compared to manual measurements across the test dataset. The scatter plot (Fig 7A) shows a moderate correlation between manual and automated ET measurements (Pearson r = 0.46, p ≈ 0.14). The Bland–Altman analysis (Fig 7B) demonstrates a small mean bias of –1.05 µm, with 95% limits of agreement from –6.46 µm to 4.37 µm. A good agreement exists for thinner epidermis of AK lesions. However, the variability of the automated measurements increases with greater thickness of epidermis, leading to lower accuracy due to underestimation in thicker regions.

## 4. Discussion

### 4.1. Key findings

This study indicates that a well-optimized, simple U-Net model can be an accurate tool for automated epidermal segmentation of AK in OCT images. Smaller input sizes (256 × 256), shorter training durations (50 epochs), and small batch sizes (2) achieved the best balance between accuracy and efficiency, while larger configurations led to overfitting. Our model reached a Dice score of 0.86, which is generally regarded as good performance in medical image segmentation [29,30]. This highlights the reliability of our model’s epidermal delineation, despite the challenges of AK imaging.

Previous studies have also shown that simpler models with shorter training durations are often more efficient for tasks such as single-layer segmentation. For instance, Derekas et al. [31] found that using moderate pixel sizes for training their modified U-Net model (AKU-Net) made segmentation more stable and efficient, leading to more accurate AK lesion delineation in clinical photographs. Similarly, Soni et al. [32] emphasized the importance of fine-tuning aspects such as image resolution and training time alongside the model architecture in their work with ARCUNet for skin lesion segmentation in dermatoscopic images.

Our study also demonstrates that small datasets can be sufficient for training lightweight U-Net models, as long as hyperparameters are well-optimized and overfitting is prevented. At this early stage, using a larger dataset could have added unnecessary variability due to differences in lesion grade or image quality, hindering the actual impact of our hyperparameter choices. On the other hand, using a small subset may provide us with a better understanding of the effects of model architecture and training decisions. Accordingly, our use of a small dataset for hyperparameter optimization is in agreement with findings from other OCT domains. Ndipenoch et al. [33] successfully trained a shallow U-Net-based model, referred to as CoNet (Coherent Network), on only 55 OCT B-scans for retinal layer and fluid segmentation, achieving robust performance comparable to human experts, demonstrating that small, high-quality datasets can be sufficient for robust performance.

Other studies have considered applying deep learning to skin imaging, but mostly in contexts that are less clinically complex than AK lesions. OCT-based segmentation in normal skin [34] and experimental models such as laser-induced injuries in mouse skin [35] achieved high accuracy, but these settings involved uniform tissue boundaries and limited variability, making segmentation relatively straightforward. The study by del Amor et al. (2020) [34], using a 2D U-Net plus post-processing on healthy human-skin OCT images, achieved a mean epidermal Dice score of 0.83 ± 0.06. An additional work by Shishkova et al. (2025) applied both 2D and 3D U-Net models to 3D OCT skin volumes from healthy humans, including Dice values of ~0.89 (2D) and ~0.87 (3D) for the epidermal cellular layer [36]. Although we used only 2D OCT B-scans, our model’s Dice score (0.859) remains very close to these two studies, highlighting that simpler 2D U-Net pipelines can perform quite well and accurately compared to 3D data or more advanced methods. AK-focused approaches using photographs or dermatoscopic images have leveraged advanced architectures, including transformer-equipped U-Nets and CNNs with ConvLSTM modules [31,37,38], yet they were constrained by the absence of depth information, low contrast, ambiguous lesion borders, and the need for manual preselection of regions. By contrast, our study addresses these limitations by using OCT to capture structural depth and a full variability of human AK lesions, demonstrating robust epidermal segmentation with a simple U-Net model.

In preclinical settings, U-Net has shown a strong segmentation performance. A study on epithelial tissue and scab segmentation in rodent skin OCT images obtained values of Dice = 0.933 and IoU ≈ 0.875, demonstrating high performance under controlled imaging and uniform tissue conditions [39]. Also, more complex hybrid architectures such as LS-Net performed better in segmentation with scores of mean Dice ≈ 0.9624 and IoU ≈ 0.9468, but with increased complexity and computational demands [40]. In this context, our proposed U-Net model (PS_256 × 256_B2_E50) achieves segmentation performance very well within the range reported in prior U-Net–based human-skin OCT studies and quite close to more complex or 3D methods. Our model remains efficient and easy to train, representing a robust and reproducible baseline for OCT epidermal segmentation, despite its simple architecture. Importantly, this high accuracy given the low technical requirements is a major advantage for clinical translation of this tool.

It is important to clarify that the primary aim of our study was not to propose a novel deep learning architecture, but rather the development and validation of a practical, clinically feasible tool for automated epidermal segmentation — a tool that can be reliably trained on a small, manually annotated OCT dataset and run on standard CPU hardware. Consequently, a key outcome of our work is to a demonstration that accurate epidermal segmentation can be performed well under these real-world constraints, achieving an applicable tool to clinical and translational settings, without requiring high-performance computing resources or large datasets.

### 4.2. Hyperparameters effects

In general, the selection of image resolution, batch size, and training epochs leads to a trade-off between training performance and generalization. Each hyperparameter tested in this study had a meaningful influence on the results. Smaller image resolutions helped the model focus and converge more quickly. The batch size affected how well the model generalized to new data, with smaller or moderate sizes yielding the best results. The number of training epochs influenced both accuracy and the risk of overfitting.

These observations are well aligned with previous studies. Lin et al. [11] showed that optimized CNN models for epidermal segmentation in OCT images benefited from smaller input sizes, which helped reduce overfitting while improving speed and focus. Their work also highlights how strategies like pixel skipping and multiprocessing can improve training efficiency without compromising segmentation quality. Soni et al. [32] also demonstrated the importance of carefully chosen epochs, especially in preventing overfitting in deeper architectures. Our results support these conclusions and add to the evidence that lightweight, well-tuned models can perform effectively in medical image segmentation.

### 4.3. Impact of AK-specific characteristics

The most challenging cases for epidermal delineation were not necessarily those with higher AK grades, but generally cases where the DEJ was difficult to identify. This ambiguity results from the fact that hyperkeratosis and other AK-associated changes may obscure the DEJ. Additionally, other skin features, such as hair strands and follicles, interrupt the DEJ and cause shadowing in the OCT images that complicates the identification of the DEJ. Consequently, even manual annotations can be inconsistent, as the same expert might delineate the DEJ differently on separate occasions. Nevertheless, our model achieved high accuracy and Dice scores, despite the variability of AK lesions and normal skin features. Likewise, Korecka et al. [41] reported difficulties in automatic AK staging from dermoscopy and high-frequency ultrasound (HFUS), particularly in advanced lesions.

### 4.4. Limitations and future work

This study is limited by a relatively small dataset restricted to AK grades 1–2 and annotations from a single expert. Broader datasets including AK lesions of grade 3 and consensus labels in skin layers would improve generalizability. Further limitations are the absence of data augmentation and the use of only central B-scans for training. However, it is noteworthy that Dice and IoU scores were comparable between central and peripheral B-scan slices, likely due to the distribution of hyperkeratosis across image locations. During manual annotation, peripheral B-scans (25% and 75% positions) had less or thinner hyperkeratosis across the image than central slices, making the DEJ easier to delineate, which may have made the DEJ easier to delineate. On the contrary, central B-slices commonly had hyperkeratosis covering a relatively small central portion of each image, while the lateral regions remained relatively well defined. Although the sample size was small, these observations may explain why segmentation performance did not differ significantly between central and peripheral slices. Nonetheless, our model performed well on the peripheral scans indicating that the central B-scan was a good representative image. Future work could expand the dataset to include additional B-scans positions, data augmentation techniques, and explore advanced deep learning models. Furthermore, while our focus on a simple U-Net architecture highlights efficiency, future work should compare with more advanced designs, including transformer-based models [31]. Extending segmentation to 3D OCT volumes could enhance robustness and lead to more comprehensive lesion monitoring, as well. Another potential limitation of our study is that we only considered one random split of the data. It might be interesting to verify our results through the implementation of multiple splits in both the training and testing processes.

Moreover, even though the automated approach of *ET* measurements shows a good overall agreement with manual segmentation, its performance is not uniform across all images. The automated segmentation provides reliable estimates for thinner lesions, whereas reduced accuracy is observed for thicker lesions. Addressing that will be a significant and promising next step, particularly to strengthen robustness in more advanced or hyperkeratotic lesions. Future research may build upon recent advances in high-resolution OCT imaging. For instance, Thamm et al. [42] demonstrated the potential of line-field confocal optical coherence tomography (LC-OCT) combined with CNNs for automated proliferation (PRO) scoring of AK lesions, achieving excellent accuracy in delineating the DEJ. Although LC-OCT provides better resolution (~ 1 µm isotropic resolution, comparable to histology), it has lower penetration depth compared to conventional OCT [43]. A promising next step will be to apply our optimized U-Net configuration to a larger longitudinal OCT dataset of AK lesions and other types of keratinocyte dysplasia, such as SCC, investigating quantitative biomarkers such as epidermal thickness and optical properties for early prognosis and treatment monitoring.

## 5. Conclusion

Our study shows that a lightweight U-Net model can effectively and efficiently segment the epidermis in OCT images of AK. After fine-tuning, we found that using an image resolution of 256 × 256 pixels with a batch size of 2 at 50 epochs yielded the best outcome, striking a balance between accuracy and speed. Notably, a simpler model works well even with a relatively small dataset, which is vital in clinical settings where time and resources are limited. Additionally, choosing the right number of training epochs was critical to avoid overfitting. The U-Net structure itself is ideal for this task because it preserves fine spatial details without adding complexity, making it excellent for precisely defining boundaries between skin layers. Our proposed model performs well under conditions that are challenging even for human observers, highlighting its robustness and clinical potential. Overall, our findings strengthen the promise of deep learning for an objective and efficient assessment of AK in OCT imaging. Finally, this model has potential to aid in differential diagnosis of SCC and the identification of imaging biomarkers of AK grade, and to aid clinicians potentially in monitoring the disease development or the therapy response.

## Supporting information

S1 Table Performance of all U-Net models on the testing set. (DOCX)

S2 Table Overview of hyperparameter configurations and their convergence or overfitting outcomes. (DOCX)

S3 Table Non-central & central B-scans evaluation for peripheral (25% and 75%) and central (50%) regions. (DOCX)

S4 Table Pairwise t-test results for peripheral and central regions. (DOCX)

S1 Fig Performance in training and validation of all models across epochs. (DOCX)

S1 File Evaluation of B-scan regions and statistical comparison. (DOCX)
