# Supplementary material for: A U-Net model for epidermal segmentation in optical coherence tomography images of actinic keratosis
Source: PLoS One. 2026 Jun 5;21(6):e0346059. doi: 10.1371/journal.pone.0346059 (PMC13240933; doi:10.1371/journal.pone.0346059)
Supplement: S1 Table — (DOCX) [file pone.0346059.s001.docx]

A U-Net model for epidermal segmentation in optical coherence tomography images of actinic keratosis

Theofanis Angelis^1, 2*^, Peter A. Philipsen^1^, Vinzent K. Ortner^1^, Gabriella Fredman^1^, Merete Haedersdal^1,3^, and Gavrielle R. Untracht^1,2^

^1^Department of Dermatology, Copenhagen University Hospital, Bispebjerg and Frederiksberg, Copenhagen, NV, 2400, Denmark

^2^Department of Health Technology, Technical University of Denmark, Kongens Lyngby, 2800, Denmark

^3^Department of Clinical Medicine, Faculty of Health and Medical Science, University of Copenhagen, Copenhagen, Denmark

^*^Corresponding author: *tangelis@outlook.com*

# Supporting Information

**S1 Table. Performance of all U-Net models on the testing set.** The best-performing values are bold and the most optimal configuration (PS_256×256_B2_E50, corresponding to image size 256×256 pixels, batch of 2 over epochs 50) is marked through the corresponding metrics. PS: image size in pixels, B: batch size, E: epochs

| Model Name | Accuracy | Precision | Recall | Jaccard Index | Dice Coefficient | Mean Absolute Error | **Hausdorff Distance** |
| --- | --- | --- | --- | --- | --- | --- | --- |
| PS_1024×1024_B16_E100 | 0.960 | 0.815 | 0.406 | 0.352 | 0.508 | 3.265 | 115.456 |
| PS_1024×1024_B16_E150 | 0.966 | 0.808 | 0.528 | 0.462 | 0.627 | 5.755 | 125.251 |
| PS_1024×1024_B16_E50 | 0.958 | 0.671 | 0.595 | 0.421 | 0.585 | 9.072 | 126.327 |
| PS_1024×1024_B2_E100 | 0.970 | 0.835 | 0.592 | 0.528 | 0.684 | 5.041 | 156.735 |
| PS_1024×1024_B2_E150 | 0.971 | 0.826 | 0.615 | 0.542 | 0.695 | 5.345 | 155.650 |
| PS_1024×1024_B2_E50 | 0.969 | 0.857 | 0.522 | 0.479 | 0.641 | 3.540 | 121.792 |
| PS_1024×1024_B4_E100 | 0.968 | 0.832 | 0.528 | 0.474 | 0.637 | 4.890 | 123.475 |
| PS_1024×1024_B4_E150 | 0.969 | 0.826 | 0.559 | 0.496 | 0.658 | 5.104 | 147.656 |
| PS_1024×1024_B4_E50 | 0.967 | 0.836 | 0.499 | 0.450 | 0.615 | 4.422 | 120.800 |
| PS_1024×1024_B8_E100 | 0.967 | 0.826 | 0.516 | 0.461 | 0.625 | 4.970 | 121.540 |
| PS_1024×1024_B8_E150 | 0.967 | 0.817 | 0.537 | 0.474 | 0.638 | 5.554 | 128.615 |
| PS_1024×1024_B8_E50 | 0.962 | 0.793 | 0.450 | 0.385 | 0.547 | 4.439 | 105.277 |
| PS_256×256_B16_E100 | 0.981 | 0.898 | 0.759 | 0.699 | 0.816 | 0.177 | 13.323 |
| PS_256×256_B16_E150 | 0.983 | 0.912 | 0.777 | 0.723 | 0.832 | 0.179 | 12.645 |
| PS_256×256_B16_E50 | 0.982 | 0.881 | 0.784 | 0.706 | 0.822 | 0.219 | 13.362 |
| PS_256×256_B2_E100 | 0.983 | 0.888 | 0.815 | 0.740 | 0.844 | 0.345 | 22.273 |
| PS_256×256_B2_E150 | 0.982 | 0.892 | 0.790 | 0.723 | 0.831 | 0.279 | 16.389 |
| PS_256×256_B2_E50 | **0.985** | 0.891 | **0.839** | **0.762** | **0.859** | 0.214 | 12.026 |
| PS_256×256_B4_E100 | 0.984 | 0.887 | 0.836 | 0.757 | 0.856 | 0.241 | **10.432** |
| PS_256×256_B4_E150 | 0.984 | 0.877 | 0.832 | 0.747 | 0.850 | 0.559 | 23.341 |
| PS_256×256_B4_E50 | 0.984 | 0.901 | 0.808 | 0.744 | 0.846 | 0.239 | 13.295 |
| PS_256×256_B8_E100 | 0.984 | 0.901 | 0.811 | 0.747 | 0.848 | 0.194 | 13.279 |
| PS_256×256_B8_E150 | 0.984 | 0.890 | 0.829 | 0.753 | 0.853 | 0.234 | 11.922 |
| PS_256×256_B8_E50 | 0.981 | **0.924** | 0.729 | 0.687 | 0.808 | **0.134** | 13.612 |
| PS_464×1356_B16_E100 | 0.980 | 0.895 | 0.735 | 0.678 | 0.800 | 0.698 | 63.284 |
| PS_464×1356_B16_E150 | 0.980 | 0.893 | 0.745 | 0.683 | 0.804 | 0.617 | 53.799 |
| PS_464×1356_B16_E50 | 0.963 | 0.865 | 0.382 | 0.361 | 0.523 | 0.489 | 60.749 |
| PS_464×1356_B2_E100 | 0.981 | 0.897 | 0.760 | 0.700 | 0.816 | 0.589 | 63.341 |
| PS_464×1356_B2_E150 | 0.982 | 0.891 | 0.772 | 0.708 | 0.821 | 0.725 | 61.779 |
| PS_464×1356_B2_E50 | 0.980 | 0.919 | 0.715 | 0.672 | 0.795 | 0.297 | 45.727 |
| PS_464×1356_B4_E100 | 0.981 | 0.904 | 0.759 | 0.704 | 0.819 | 0.465 | 55.714 |
| PS_464×1356_B4_E150 | 0.981 | 0.901 | 0.758 | 0.702 | 0.817 | 0.515 | 55.578 |
| PS_464×1356_B4_E50 | 0.980 | 0.914 | 0.717 | 0.671 | 0.796 | 0.358 | 47.673 |
| PS_464×1356_B8_E100 | 0.980 | 0.899 | 0.740 | 0.683 | 0.804 | 0.517 | 50.686 |
| PS_464×1356_B8_E150 | 0.981 | 0.907 | 0.739 | 0.688 | 0.807 | 0.475 | 51.528 |
| PS_464×1356_B8_E50 | 0.979 | 0.893 | 0.726 | 0.668 | 0.793 | 0.456 | 48.749 |
| PS_512×512_B16_E100 | 0.980 | 0.892 | 0.750 | 0.687 | 0.808 | 0.853 | 53.262 |
| PS_512×512_B16_E150 | 0.980 | 0.905 | 0.727 | 0.675 | 0.799 | 0.621 | 49.257 |
| PS_512×512_B16_E50 | 0.968 | 0.895 | 0.496 | 0.461 | 0.623 | 0.607 | 37.870 |
| PS_512×512_B2_E100 | 0.982 | 0.893 | 0.787 | 0.722 | 0.831 | 0.859 | 62.773 |
| PS_512×512_B2_E150 | 0.982 | 0.874 | 0.794 | 0.713 | 0.826 | 1.462 | 70.533 |
| PS_512×512_B2_E50 | 0.982 | 0.904 | 0.760 | 0.704 | 0.819 | 0.538 | 51.338 |
| PS_512×512_B4_E100 | 0.982 | 0.911 | 0.759 | 0.708 | 0.822 | 0.453 | 53.911 |
| PS_512×512_B4_E150 | 0.982 | 0.895 | 0.782 | 0.717 | 0.829 | 0.912 | 62.009 |
| PS_512×512_B4_E50 | 0.981 | 0.906 | 0.747 | 0.692 | 0.811 | 0.432 | 41.207 |
| PS_512×512_B8_E100 | 0.981 | 0.915 | 0.743 | 0.695 | 0.813 | 0.338 | 41.322 |
| PS_512×512_B8_E150 | 0.981 | 0.912 | 0.734 | 0.687 | 0.807 | 0.446 | 44.526 |
| PS_512×512_B8_E50 | 0.980 | 0.902 | 0.721 | 0.668 | 0.794 | 0.528 | 42.022 |
