# Supplementary material for: A U-Net model for epidermal segmentation in optical coherence tomography images of actinic keratosis
Source: PLoS One. 2026 Jun 5;21(6):e0346059. doi: 10.1371/journal.pone.0346059 (PMC13240933; doi:10.1371/journal.pone.0346059)
Supplement: S2 Table — (DOCX) [file pone.0346059.s002.docx]

A U-Net model for epidermal segmentation in optical coherence tomography images of actinic keratosis

Theofanis Angelis^1, 2*^, Peter A. Philipsen^1^, Vinzent K. Ortner^1^, Gabriella Fredman^1^, Merete Haedersdal^1,3^, and Gavrielle R. Untracht^1,2^

^1^Department of Dermatology, Copenhagen University Hospital, Bispebjerg and Frederiksberg, Copenhagen, NV, 2400, Denmark

^2^Department of Health Technology, Technical University of Denmark, Kongens Lyngby, 2800, Denmark

^3^Department of Clinical Medicine, Faculty of Health and Medical Science, University of Copenhagen, Copenhagen, Denmark

^*^Corresponding author: *tangelis@outlook.com*

# Supporting Information

**S2 Table**. **Overview of hyperparameter configurations and their convergence or overfitting outcomes.**

| **Image Size** | **Batch Size** | **Epochs** | **Converged (Y/N)**  **yes / no** | **Actual Epochs Completed** | **Overfitting Notes** |
| --- | --- | --- | --- | --- | --- |
| 1024×1024 | 2 | 50 | Y | 50 | None |
| 1024×1024 | 2 | 100 | Y | 100 | None |
| 1024×1024 | 2 | 150 | Y | 150 | None |
| 1024×1024 | 4 | 50 | Y | 50 | None |
| 1024×1024 | 4 | 100 | Y | 100 | None |
| 1024×1024 | 4 | 150 | Y | 150 | None |
| 1024×1024 | 8 | 50 | Y | 50 | None |
| 1024×1024 | 8 | 100 | Y | 100 | None |
| 1024×1024 | 8 | 150 | Y | 150 | None |
| 1024×1024 | 16 | 50 | Y | 50 | Some overfitting observed |
| 1024×1024 | 16 | 100 | Y | 53 | Some overfitting observed |
| 1024×1024 | 16 | 150 | Y | 150 | Some overfitting observed |
| 256×256 | 2 | 50 | Y | 50 | None |
| 256×256 | 2 | 100 | Y | 100 | None |
| 256×256 | 2 | 150 | Y | 150 | None |
| 256×256 | 4 | 50 | Y | 50 | None |
| 256×256 | 4 | 100 | Y | 100 | None |
| 256×256 | 4 | 150 | Y | 150 | None |
| 256×256 | 8 | 50 | Y | 50 | None |
| 256×256 | 8 | 100 | Y | 100 | None |
| 256×256 | 8 | 150 | Y | 150 | None |
| 256×256 | 16 | 50 | Y | 50 | None |
| 256×256 | 16 | 100 | Y | 100 | None |
| 256×256 | 16 | 150 | Y | 150 | Some overfitting observed |
| 464×1356 | 2 | 50 | Y | 50 | None |
| 464×1356 | 2 | 100 | Y | 100 | None |
| 464×1356 | 2 | 150 | Y | 150 | None |
| 464×1356 | 4 | 50 | Y | 50 | None |
| 464×1356 | 4 | 100 | Y | 100 | None |
| 464×1356 | 4 | 150 | Y | 150 | None |
| 464×1356 | 8 | 50 | Y | 50 | None |
| 464×1356 | 8 | 100 | Y | 100 | None |
| 464×1356 | 8 | 150 | Y | 150 | None |
| 464×1356 | 16 | 50 | N | 47 | None |
| 464×1356 | 16 | 100 | Y | 100 | Some overfitting observed |
| 464×1356 | 16 | 150 | Y | 150 | None |
| 512×512 | 2 | 50 | Y | 50 | None |
| 512×512 | 2 | 100 | Y | 100 | None |
| 512×512 | 2 | 150 | Y | 150 | None |
| 512×512 | 4 | 50 | Y | 50 | None |
| 512×512 | 4 | 100 | Y | 100 | None |
| 512×512 | 4 | 150 | Y | 150 | None |
| 512×512 | 8 | 50 | Y | 50 | None |
| 512×512 | 8 | 100 | Y | 100 | None |
| 512×512 | 8 | 150 | Y | 150 | None |
| 512×512 | 16 | 50 | N | 42 | Some overfitting observed |
| 512×512 | 16 | 100 | Y | 100 | Some overfitting observed |
| 512×512 | 16 | 150 | Y | 150 | Some overfitting observed |
