# Supplementary material for: A U-Net model for epidermal segmentation in optical coherence tomography images of actinic keratosis
Source: PLoS One. 2026 Jun 5;21(6):e0346059. doi: 10.1371/journal.pone.0346059 (PMC13240933; doi:10.1371/journal.pone.0346059)
Supplement: S3 Table — (DOCX) [file pone.0346059.s003.docx]

A U-Net model for epidermal segmentation in optical coherence tomography images of actinic keratosis

Theofanis Angelis^1, 2*^, Peter A. Philipsen^1^, Vinzent K. Ortner^1^, Gabriella Fredman^1^, Merete Haedersdal^1,3^, and Gavrielle R. Untracht^1,2^

^1^Department of Dermatology, Copenhagen University Hospital, Bispebjerg and Frederiksberg, Copenhagen, NV, 2400, Denmark

^2^Department of Health Technology, Technical University of Denmark, Kongens Lyngby, 2800, Denmark

^3^Department of Clinical Medicine, Faculty of Health and Medical Science, University of Copenhagen, Copenhagen, Denmark

^*^Corresponding author: *tangelis@outlook.com*

# Supporting Information

**S3 Table. Non-central & central B-scans evaluation** for peripheral (25% and 75%) and central (50%) regions.

| **Subset** | **Dice** | **IoU** | **Precision** | **Recall** | **Accuracy** |
| --- | --- | --- | --- | --- | --- |
| 25% | 0.55 | 0.38 | 1.00 | 0.38 | 0.94 |
| 25% | 0.53 | 0.36 | 0.84 | 0.39 | 0.96 |
| 25% | 0.59 | 0.41 | 0.56 | 0.61 | 0.97 |
| 25% | 0.67 | 0.50 | 0.72 | 0.62 | 0.97 |
| 25% | 0.65 | 0.49 | 0.67 | 0.64 | 0.97 |
| 50% | 0.61 | 0.44 | 0.94 | 0.46 | 0.96 |
| 50% | 0.67 | 0.50 | 0.89 | 0.54 | 0.98 |
| 50% | 0.60 | 0.43 | 0.73 | 0.51 | 0.97 |
| 50% | 0.67 | 0.51 | 0.70 | 0.65 | 0.97 |
| 50% | 0.64 | 0.47 | 0.59 | 0.70 | 0.97 |
| 75% | 0.53 | 0.36 | 1.00 | 0.36 | 0.94 |
| 75% | 0.66 | 0.50 | 0.70 | 0.63 | 0.97 |
| 75% | 0.61 | 0.44 | 0.93 | 0.46 | 0.96 |
| 75% | 0.63 | 0.46 | 0.74 | 0.54 | 0.97 |
| 75% | 0.65 | 0.49 | 0.67 | 0.64 | 0.97 |
