# Supplementary material for: A U-Net model for epidermal segmentation in optical coherence tomography images of actinic keratosis
Source: PLoS One. 2026 Jun 5;21(6):e0346059. doi: 10.1371/journal.pone.0346059 (PMC13240933; doi:10.1371/journal.pone.0346059)
Supplement: S4 Table — (DOCX) [file pone.0346059.s004.docx]

A U-Net model for epidermal segmentation in optical coherence tomography images of actinic keratosis

Theofanis Angelis^1, 2*^, Peter A. Philipsen^1^, Vinzent K. Ortner^1^, Gabriella Fredman^1^, Merete Haedersdal^1,3^, and Gavrielle R. Untracht^1,2^

^1^Department of Dermatology, Copenhagen University Hospital, Bispebjerg and Frederiksberg, Copenhagen, NV, 2400, Denmark

^2^Department of Health Technology, Technical University of Denmark, Kongens Lyngby, 2800, Denmark

^3^Department of Clinical Medicine, Faculty of Health and Medical Science, University of Copenhagen, Copenhagen, Denmark

^*^Corresponding author: *tangelis@outlook.com*

# Supporting Information

**S4 Table. Pairwise t-test results for peripheral and central regions.**

| **Metric** | **Mean_Peripheral (25%)** | **Mean_Central (50%)** | **Mean_Peripheral (75%)** | **t_25_75** | **p_25_75** | **t_50_75** | **p_50_75** |
| --- | --- | --- | --- | --- | --- | --- | --- |
| Dice | 0.60 | 0.64 | 0.62 | -0.61 | 0.58 | 1.28 | 0.27 |
| IoU | 0.43 | 0.47 | 0.45 | -0.69 | 0.53 | 1.04 | 0.36 |
