# Supplementary material for: A U-Net model for epidermal segmentation in optical coherence tomography images of actinic keratosis
Source: PLoS One. 2026 Jun 5;21(6):e0346059. doi: 10.1371/journal.pone.0346059 (PMC13240933; doi:10.1371/journal.pone.0346059)
Supplement: S1 Fig — (DOCX) [file pone.0346059.s005.docx]

A U-Net model for epidermal segmentation in optical coherence tomography images of actinic keratosis

Theofanis Angelis^1, 2*^, Peter A. Philipsen^1^, Vinzent K. Ortner^1^, Gabriella Fredman^1^, Merete Haedersdal^1,3^, and Gavrielle R. Untracht^1,2^

^1^Department of Dermatology, Copenhagen University Hospital, Bispebjerg and Frederiksberg, Copenhagen, NV, 2400, Denmark

^2^Department of Health Technology, Technical University of Denmark, Kongens Lyngby, 2800, Denmark

^3^Department of Clinical Medicine, Faculty of Health and Medical Science, University of Copenhagen, Copenhagen, Denmark

^*^Corresponding author: *tangelis@outlook.com*

# Supporting Information





**S1 Fig. Performance in training and validation of all models across epochs.** Image plots of training accuracy (top left), validation accuracy (top right), training loss (bottom left), and validation loss (bottom right). The U-Net model of image size 256×256 pixels and batch of 2 at epochs 50 is provided in red and bold, to indicate that this model has outperformed all other suitable models in the plot.
