# Supplementary material for: A U-Net model for epidermal segmentation in optical coherence tomography images of actinic keratosis
Source: PLoS One. 2026 Jun 5;21(6):e0346059. doi: 10.1371/journal.pone.0346059 (PMC13240933; doi:10.1371/journal.pone.0346059)
Supplement: S1 File — (DOCX) [file pone.0346059.s006.docx]

A U-Net model for epidermal segmentation in optical coherence tomography images of actinic keratosis

Theofanis Angelis^1, 2*^, Peter A. Philipsen^1^, Vinzent K. Ortner^1^, Gabriella Fredman^1^, Merete Haedersdal^1,3^, and Gavrielle R. Untracht^1,2^

^1^Department of Dermatology, Copenhagen University Hospital, Bispebjerg and Frederiksberg, Copenhagen, NV, 2400, Denmark

^2^Department of Health Technology, Technical University of Denmark, Kongens Lyngby, 2800, Denmark

^3^Department of Clinical Medicine, Faculty of Health and Medical Science, University of Copenhagen, Copenhagen, Denmark

^*^Corresponding author: *tangelis@outlook.com*

# **Supporting Information**

**S1 File.** **Evaluation of B-scan regions and statistical comparison**

**We evaluated both central and peripheral B-scans regions. As we can see in S3 Table across the subsets, precision, recall, and accuracy are generally high in all regions, while Dice and IoU scores are slightly higher in the central region (50%) compared to the peripheral regions (25% and 75%). Hence,** we performed pairwise t-test on both peripheral and central regions for Dice and IoU mean scores (S4 Table). In the t-tests, a positive t-value indicates that the first group performed slightly better, while a negative t-value indicates slightly worse performance. Although the central B-scans were used for training, the differences in Dice and IoU compared with peripheral slices are **not statistically significant (all p > 0.05)**. This indicates that the model’s segmentation performance **generalizes well to non-central slices**, supporting its robustness along the slow axis of the lesion**.**
